# The Utility of Genomic Testing for Hyperphenylalaninemia

**DOI:** 10.3390/jcm11041061

**Published:** 2022-02-18

**Authors:** Elisabetta Anna Tendi, Maria Guarnaccia, Giovanna Morello, Sebastiano Cavallaro

**Affiliations:** Institute for Biomedical Research and Innovation, National Research Council, Via Paolo Gaifami 18, 95026 Catania, Italy; elisabetta.tendi@irib.cnr.it (E.A.T.); maria.guarnaccia@cnr.it (M.G.); giovanna.morello@irib.cnr.it (G.M.)

**Keywords:** hyperphenylalaninemia, genomics, inherited metabolic disorders

## Abstract

Hyperphenylalaninemia (HPA), the most common amino acid metabolism disorder, is caused by defects in enzymes involved in phenylalanine metabolism, with the consequent accumulation of phenylalanine and its secondary metabolites in body fluids and tissues. Clinical manifestations of HPA include mental retardation, and its early diagnosis with timely treatment can improve the prognosis of affected patients. Due to the genetic complexity and heterogeneity of HPA, high-throughput molecular technologies, such as next-generation sequencing (NGS), are becoming indispensable tools to fully characterize the etiology, helping clinicians to promptly identify the exact patients’ genotype and determine the appropriate treatment. In this review, after a brief overview of the key enzymes involved in phenylalanine metabolism, we represent the wide spectrum of genes and their variants associated with HPA and discuss the utility of genomic testing for improved diagnosis and clinical management of HPA.

## 1. Introduction

A burden of phenylalanine (Phe) in the blood and other tissues is the hallmark of hyperphenylalaninemia (HPA), the most common inborn error of amino acid metabolism, with an incidence that varies widely among ethnic and geographical regions around the world [1,2]. HPA is often the result of genetic alterations in the phenylalanine hydroxylase (*PAH*) gene, encoding an enzyme catalyzing the conversion of L-Phe to L-Tyrosine (Tyr), but it may also derive from defects in genes encoding enzymes involved in the biosynthesis or regeneration of the cofactor tetrahydrobiopterin (BH4) [3]. Although HPA is primarily characterized by progressive mental retardation, distinctive genotypes associated with HPA have different effects on the severity and prognosis of the disease and the response of patients to therapy [4,5]. To avoid irreversible damage to the nervous system, it is essential to perform an early and accurate diagnosis and begin the appropriate treatment in a timely manner.

To date, many countries in the world have implemented newborn screening (NBS) programs that allow the diagnosis of HPA and elicit a prompt therapy, which is often based on a diet throughout life [6,7]. However, traditional differential diagnosis methods are time consuming and are nowadays inadequate to capture the extensive genetic heterogeneity of HPA. In this context, high-throughput technologies, such as multiplex ligation-dependent probe amplification, DNA microarray and next-generation sequencing (NGS), allow the simultaneous analysis of multiple genetic variants associated with this heterogeneous disorder and, thus, optimize patient care and management [8,9,10,11,12,13].

In this review, after a brief overview of the key enzymes involved in Phe metabolism, we represent the wide spectrum of genes and their variants associated with HPA and discuss the utility of genomic testing for improved diagnosis and clinical management of HPA. To introduce the readers to genomic testing, we exemplify the workflow and illustrate time and cost of targeted NGS applied to HPA.

## 2. Enzymes Involved in Phe Metabolism

**Figure 1:**
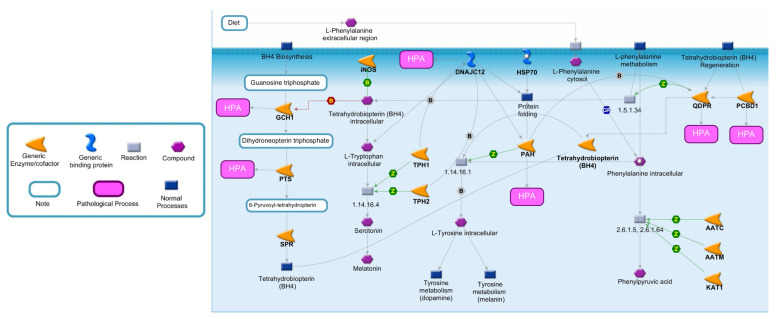
Metabolic pathway of Phe and BH4. Phe metabolism is crucial for protein synthesis, as well as for the synthesis of Tyr and its derivatives. The major catabolic pathway involves Phe hydroxylation to Tyr by PAH. In one of the minor pathways, Phe may undergo conversion to Phenylpyruvic acid. An essential cofactor and regulator of PAH is BH4, whose biosynthesis and regeneration depend by different enzymes. Abbreviations: AATC, Aspartate aminotransferase, cytoplasmic; AATM, Aspartate aminotransferase, mitochondrial; DCoH, Pterin-4-alpha-carbinolamine dehydratase; DHPR, Dihydropteridine reductase; DNAJC12, DNAJ homolog subfamily C member 12; GCH1, Guanosine triphosphate (GTP) cyclohydrolase 1; KAT1, Kynurenine-oxoglutarate transaminase 1; PAH, Phenylalanine-4-hydroxylase; PTS, 6-pyruvoyl tetrahydrobiopterin synthase; SPR, Sepiapterin reductase; TPH1, Tryptophan 5-hydroxylase 1; TPH2, Tryptophan 5-hydroxylase 2; PCBD1, Pterin-4a-carbinolamine dehydratase; EC number 1.5.1.34: Dihydrobiopterin + NADH = NAD^+^ + Tetrahydrobiopterin; EC number 1.14.16.1: O_2_ + Tetrahydrobiopterin + S-Methylcysteine = S-Methylcysteine-sulfoxide + H_2_O + Dihydrobiopterin; EC number: 1.14.16.4 L-Tryptophan + O_2_ + Tetrahydrobiopterin = 5-Hydroxyl-L-Tryptophan+4alpha-Hydroxytetrahydrobiopterin; EC number 2.6.1.5-2.6.1.64: L-Phenylalanine + 2-Oxoglutaric acid = L-Glutamic acid + Phenylpyruvic acid.

To better highlight the effects of enzymatic deficiencies on Phe metabolism, all the enzymes directly or indirectly involved in Phe catabolism are illustrated in Figure 1 [14]. Phe is an essential amino acid exclusively obtained by the diet or by endogenous proteolysis. Following cellular exogenous uptake through specific transporters, Phe is converted in L-Tyr by PAH, an enzyme mainly expressed in the liver and kidney, which represents the rate-limiting step in Phe catabolism [15,16,17]. This conversion is dependent on tetrahydrobiopterin (BH4), as a cofactor, molecular oxygen and iron [18]. When PAH is nonfunctional, Phe accumulates in the body and is converted by different enzymes into phenylpyruvic acid, a substance that is normally produced only in small quantities. Individuals with mutations of *PAH* excrete in the urine large quantities of phenylpyruvic acid along with Phe, a condition known as phenylketonuria (PKU) [19].

PAH activity depends on BH4, a cofactor also involved in the hydroxylation of intracellular tryptophan (a precursor of serotonin and melatonin) and Tyr (a precursor of dopamine and melanin), as well as the synthesis of nitric oxide synthase and the cleavage of lipid ethers into glycerol and the corresponding aldehyde [20,21]. As illustrated in Figure 1, under normal conditions, the de novo biosynthesis of BH4 from guanosine triphospate (GTP) is catalyzed by three enzymes: GTP cyclohydrolase I (GCH1), 6-pyruvoyl-tetrahydropterin synthase (PTS) and sepiapterin reductase (SPR) [18,22,23]. An alternative or salvage pathway involves the regeneration of BH4 from dihydrobiopterin by pterin-4-alpha-carbinolamine dehydratase (PCBD1) and quinoid dihydropteridine reductase (QDPR) [24]. Although the major controlling point in BH4 biosynthesis is GCH1, defects in all enzymes, with the exception of SPR, can be a cause of HPA.

The proper folding and degradation of PAH is regulated by DNAJC12, a member of the subclass of the DNAJ/Hsp40 family of cochaperones, which modulate the activity of molecular chaperone Hsp70 [25]. In particular, DNAJC12 directly interacts with PAH and may play a role in the Hsp70-assisted folding of PAH and in the processing of misfolded ubiquitinated PAH [26]. The deficiency of DNAJC12 leads to decreased PAH protein levels and activity [27].

## 3. Genetics of Hyperphenylalaninemia

In Table 1, we list all the genes associated with HPA. About 98% of cases are caused by loss-of-function mutations in *PAH* that, as described before, encodes the enzyme performing the rate-limiting step in Phe catabolism. In a few cases, HPA is associated with mutations of *DNAJC12*, whose encoded protein controls proper folding and degradation of PAH [26,27]. In the remaining cases, HPA originates from defects in genes encoding enzymes involved in the biosynthesis (GCH1, PTS) or regeneration (PCBD1, QDPR) of BH4, the active cofactor of PAH [28]. Below is a description of each of these genes and their allelic variants.

HPA derived from mutations of *PAH* (chromosome 12q23.2, 13 exons) shows an autosomal recessive (AR) inheritance (Table 1). To date, more than 1000 mutations have been described and reported in the locus-specific *PAH* database (http://www.biopku.org, accessed on 23 December 2021), including single-nucleotide variants (SNVs), short insertions and deletions (InDels) and large structural variants (SVs) [29,30,31].

*DNAJC12* (chromosome 10q21.3, 6 exons), also known as *JDP1* or *HPANBH4*, encodes for a heat shock co-chaperone family member protein involved in proper folding of PAH [32]. The destabilization of this enzyme caused by AR mutations with subsequent loss of Phe, Tyr and neuronal tryptophan hydroxylases activity, leads to HPA and neurotransmitter deficiency [33,34] (Table 1). To date, different pathogenic or likely pathogenic variants have been associated with mild and non-BH4-deficient HPA, causing nonsense, frameshift, missense and splice-site mutations [35,36]. Recently, new heterozygous mutations in *DNAJC12* were found by whole exome sequencing (WES), further supporting the importance of high-throughput screening methods for discovering and improving the neurodevelopmental outcome of HPA patients [32].

*GCH1* (chromosome 14q22.2, 7 exons) encodes the first and rate-limiting enzyme of BH4 biosynthesis [18]. Its deficiency causes DOPA-responsive dystonia with or without HPA [37]. The most common dominant form, known as Segawa disease, responds well to dopamine replacement therapy, whereas the recessive form is more severe and is associated with malignant HPA [38,39,40]. In some patients with autosomal recessive *GCH1* deficiency, the diagnosis can be late due to normal blood phenylalanine levels at NBS [41]. Different pathogenic *GCH1* variants are known for producing a variety of molecular consequences (Table 1).

*PTS* (chromosome 11q23.1, 6 exons) encodes 6-pyruvoyl-tetrahydropterin synthase, an enzyme involved in the catalytic conversion of dihydroneopterin triphosphate to 6-pyruvoyl-tetrahydropterin and elimination of inorganic triphosphate from dihydroneopterin triphosphate, which is the second and irreversible step in the biosynthesis of BH4 [42,43]. Autosomal recessive genetic variations in *PTS*, which account for approximately 60% of all BH4 deficiencies, are associated with severe or mild forms of HPA [11,44]. Deletions, duplications, insertion and single nucleotide *PTS* variants (Table 1) can result in decreased or null enzyme activity, thus leading to little or no BH4 production and consequently to toxic levels of Phe in blood and other tissues [42].

*PCBD1* (chromosome 10q22.1, 6 exons) encodes for Pterin-4 Alpha-Carbinolamine Dehydratase 1, an enzyme involved in the regeneration of BH4 whose defects are associated with a benign transient form of HPA [45]. Different *PCBD1* variants (missense, frameshift, nonsense, and small deletions in exon 2 and 4) may reduce enzyme activity and result in pathogenic effects [39,46] (Table 1).

*QDPR* (chromosome 4p15.32, 7 exons) encodes the enzyme quinoid dihydropteridine reductase, catalyzing the regeneration of BH4 from quinonoid dihydropteridine (qBH2) [47]. Deficiency of *QDPR* causes an atypical PKU form due to the insufficient production of BH4 associated with severe neurological deterioration, microcephalia, psychomotor retardation, delayed development, tonal abnormalities, myoclonic epilepsy and dystonia [48]. Several pathogenic or likely pathogenic *QDPR* variants have been described [49] (Table 1).

## 4. Differential Diagnosis

Deficiencies in PAH or its cofactor BH4 can affect Phe homeostasis and lead to HPA. Most of the clinical manifestations associated with HPA are attributable to the increased levels of Phe and the depletion of monoamine neurotransmitters in the central nervous system [50]. The precise and early diagnosis of HPA represents the most important goal to avoid its harmful effects [51]. Indeed, the progressive neurologic manifestations, which include movement disorders, seizures, mental retardation, dyskinesias, microcephaly and hyperthermia, can be prevented or reduced with the choice of an early diagnosis and the right therapy [2,52]. The first step in the diagnostic strategy is the definition of the HPA subtypes. HPA with diverse severity degrees can be distinguished by different circulating blood Phe levels (a value up to 120 μmol/L is considered normal), response to diet and type of impaired enzymatic activity [53]. Specifically, patients with HPA can be classified as classic PKU (>1200 μM), moderate PKU (900–1200 μM), mild PKU (600–900 μM), mild HPA (<600 μM) or BH4 deficiency [54,55,56].

Tests used to diagnose and monitor patients with various degrees of severity of HPA include the quantification of Phe and Tyr concentrations by tandem mass spectrometry, the evaluation of pterin concentrations (neopterin, biopterin, primapterin, anapterin, and 6-oxo-primapterin) in urine or blood, the evaluation of PAH enzymatic activity in liver and kidney tissues, and the use of molecular genetic assays to screen for pathogenic variants in genes involved in HPA [57,58]. The latter is performed in infants with high levels of Phe and mainly involves genetic tests for *PAH* and/or other genes involved in the Phe metabolic pathway [59]. *PAH* mutations vary in their consequences for the residual level of PAH activity, from having little or no effect to abolishing PAH activity completely [60,61]. Once HPA is diagnosed at an early stage, the use of a specific diet can help to reduce the clinical outcomes of this disease. The use of BH4, alone or in addition to diet, can be used to further lower elevated blood Phe levels [3]. To this regard, sapropterin dihydrochloride (Kuvan, BioMarin Pharmaceutical Inc.) represents an orally active synthetic form of BH4 effective therapy that can be used in selected patients with HPA and mild-to-moderate PKU following a BH4 loading test [62,63].

Although biochemical NBS tests represent reliable diagnostic tools, they do not allow to identify the causes responsible for high Phe levels, which may be also transient and related to different factors, such as medical therapies, prematurity, liver metabolic immaturity, and parenteral nutrition [64]. Moreover, NBS tests are restricted to a limited number of metabolites associated with PHA, while the Phe metabolic pathway (Figure 1) is complex and involves additional key mediators with enzymatic, transporter or regulatory functions [14]. Since different metabolic phenotypes of HPA exist and depend upon variations in six different genes (Table 1), reaching a precise differential diagnosis and classification is not easy. To this end, genetic testing represents a suitable approach for a better genotype/phenotype correlation and, hopefully, improving the development of future innovative therapeutic interventions, such as gene therapy [65].

## 5. Current and Future Therapy for HPA

Without effective treatments, most people with HPA would develop neurological manifestations, among which intellectual disability is the most severe form [66]. To prevent neurological injuries, the mainstay of treatment for PAH deficiency consists of a carefully controlled Phe-restricted diet beginning the first days or weeks of life [67]. When diet therapy starts in early childhood, it helps to prevent the main manifestations of this metabolic disorder, although this treatment may not be as effective as the patient has to follow a complicated and unpleasant diet throughout the life. Adherence to dietary therapy in adolescents and adults is poor with up to 85–90% of patients exhibiting blood Phe concentrations above target levels [68,69]. Consequently, it is easy to assist to the development of a range of unsatisfactory outcomes, including neuropsychiatric symptoms [70]. The need to evaluate innovative therapies against HPA led researchers to investigate new ways to deal with this metabolic disorder, searching for new treatments that are not strictly dependent on dietary protein restriction. One of these is the gene correction strategy, which replaces defective genes with healthy ones and represents an attractive approach to the treatment of genetic diseases [71]. Thanks to the advent of new gene therapy technologies, such as the clustered regularly interspaced short palindromic repeats (CRISPR)-Cas9 system, which has revolutionized the field of molecular biology and medicine, the chance to cure genetic disorders such as HPA may not be far away [72,73,74,75,76,77,78,79,80,81,82]. In the perspective of gene therapy for HPA, its comprehensive genomic assessment will be necessary to group patients into diagnostic, prognostic or therapeutical clusters.

## 6. Genomic Testing to Improve the Management of HPA

In recent years, we have witnessed a new revolution in genetic testing, made possible by the fields of genomics and high-throughput technologies [83]. The field of genomics has evolved into a powerful approach to gain new biological insights, study the molecular pathways underlying health and disease, and the interaction between genes to find new approaches for the diagnosis, care delivery and development of therapies [84,85]. Based on these advances, we believe that genomic testing is not only useful in HPA, where the underlying causes are a number of genes and associated variants [86], but is nowadays feasible for clinical use [87,88]. Table 2 shows the advantages and challenges of various high-throughput methods. Among these, NGS represents the most powerful tool that may rapidly and effectively analyze HPA-associated genes, providing accurate results with a faster turnaround and a lower cost than traditional methods [89,90]. NGS-based targeted gene panels (TGPs) are particularly ideal for analyzing specific mutations or genes associated with HPA [11]. They offer greater coverage of selected regions of interest, faster turnaround time, and more clinically relevant data compared to broader genomic profiling, such as WES or whole genome sequencing (WGS) approaches [91] or CGH Microarray analysis [8,9,10,11]. The advantages of using TGPs are many: (i) they can be customized for different sample types and specific genomic regions of interest; (ii) the use of lower input amounts (1 ng compared to 100 ng required for WES); (iii) the possibility to identify rare variants; (iv) a workflow simpler and shorter than WES; (v) the possibility to process thousands of samples in a single sequencing run; and (vi) a minor cost than WGS, WES or CGH microarray analysis [92,93] (Table 2).

In HPAs, the use of NGS-based TGP technology to search for new or rare variants may bring out a hitherto unexplored complexity and help to explain atypical phenotypes [94]. Back in 2014, Trujillano et al. showed that shifting from Sanger methods to high-throughput targeted resequencing improves differential diagnosis of HPA and produces a quicker establishment of specifically tailored treatments. The benefits also include a 60–80% cost savings per sample and a faster diagnostic process compared to traditional techniques [11]. In the same year, Y. Cao et al. used a customized NGS-based panel to detect mutations in HPA-related genes (*PAH*, *PTS*, *QDPR*, *GCH1*, and *PCBD1*), which provided a broader coverage, higher throughput, and a faster and more efficient solution compared with traditional molecular methods [95]. A 2017 study demonstrated the successful use of NGS to detect known and novel (one in *PAH* and two in *PTS*) causative mutations in PKU and BH4-deficiency cases, enabling accurate diagnosis and the appropriate effective treatment of patients [96,97].

**Figure 2:**
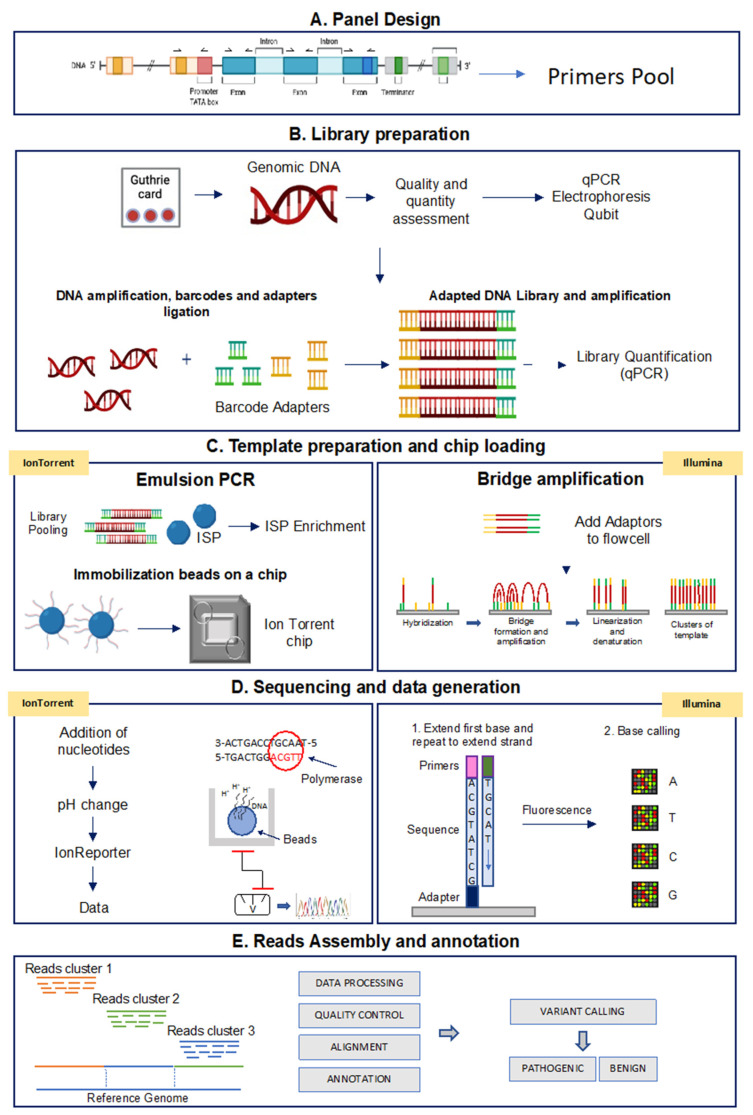
Analytical workflow of targeted sequencing for the Ion Torrent and Illumina methods. (**A**) Panel design: the Designer software helps to create custom assays based on PCR target selection. (**B**) Library preparation: library construction is the preparation of the nucleic acid target into a form compatible with the sequencing system to be used. (**C**) Template preparation and chip loading: target enrichment is used in NGS workflows to capture only genomic DNA regions of interest. (**D**) Sequencing and data generation: IonTorrent platform: microwells of the chip is flooded by nucleotides that when binding to the complementary nucleotide on a template, release an ion. At each flow, the electrical signal at each well is measured, indicating that a reaction has occurred; Illumina platform: the fragments are clonally amplified on the slide utilizing fluorescently labeled reversible-terminator nucleotides; (**E**) Read assembly and annotation: starting from Binary Alignment Map (BAM) and Variant Call Format (VCF) files, variants are prioritized based on localization, functional effect, mode of inheritance, coverage and Minor Allelic Frequency (MAF) to obtain disease-correlated variants.

Although different NGS platforms have been implemented, all NGS methods include steps performed on the laboratory bench (“wet bench”) and data analyses performed with bioinformatics pipelines (“dry bench”) [98,99]. Figure 2 shows a schematic representation of the NGS-based TGPs workflow performed with the Ion Torrent and the Illumina technologies. 

In the next sections, we represent the main steps of a TGP-based NGS analysis.

Panel design: a custom panel can be designed using the Ion AmpliSeq Designer Tool for Ion Torrent platform (Thermo Fisher Scientific) and the DesignStudio Sequencing Assay Designer for Illumina, and information contained in the NCBI (National Center for Biotechnology Information) ClinVar reference databases can be used to identify the clinical relevance of the identified variants. The two Designer tools allow the easy selection of genes ID or chromosomal coordinates across scientifically curated gene sets. The number of primers depends on the complexity and size of the genomic region to be analyzed. To estimate the number of samples that can be sequenced in multiplex assays, users need to consider different parameters, such as expected sequencing coverage and used chip type. In general, a 30× minimum coverage is recommended for germline detection mutations. Table 3 shows the key features of a custom panel created with both the AmpliSeq Designer Tool for the Ion Torrent platform and the DesignStudio Sequencing Assay Designer for the Illumina platform to analyze the genes associated with HPA. As indicated, less than 60 amplicons are needed to screen a total of 6 HPA related genes, with a 100% coverage per single amplicon.

Library preparation: the first step of NGS-based TGPs workflow (Figure 2) involves library preparation. Genomic DNA is PCR amplified with the designed panel primers (see above) and then specific barcode adapters are incorporated to allow the later clonal amplification of libraries and the identification of each sample read after the pooling of libraries.

Template preparation and chip loading: following library quantification and normalization, the libraries can be pooled and used for template preparation. In this step, using the Ion Torrent platform, an emulsion-based PCR-amplification of each amplicon is performed around Ion Sphere Particles (ISPs) containing a primer complementary to one of the adapters added during the library preparation. When the concentration of the libraries is optimized, one sample amplicon is amplified around each ISP (clonal amplification). As a final step, the DNA strands are separated, and the single strands anchored to the ISP are ready to be loaded on the microwells of the semiconductor Ion Chip. In the Illumina platform, once the DNA is amplified and the adapters are added, the modified DNA is loaded onto a flow cell where the amplification and sequencing take place. The flow cell contains nanowells that space out fragments and help with overcrowding. Each nanowell contains oligonucleotides that provide an anchoring point for the adapters to attach. Once the fragments are attached, a phase called cluster generation begins. This step makes about a thousand copies of each fragment of DNA and it is performed by bridge amplification PCR.

Sequencing and data analysis: in this step, when using the Ion Torrent platform, microwells are flooded with a single species of deoxyribonucleotide triphosphate (dNTP). If the introduced dNTP is incorporated into the growing complementary strand, the release of a hydrogen ion triggers an ISFET ion sensor, which indicates that a reaction has occurred. The series of electrical pulses transmitted from each microwell of the chip to a computer is translated real time into a DNA sequence, which is then aligned to a genome and analyzed for the presence of variants. The Illumina platform, instead, adopts a sequencing-by-synthesis approach, utilizing fluorescently labeled reversible-terminator nucleotides, on clonally amplified DNA templates (bridge amplification) immobilized to an acrylamide coating on the surface of a glass flow cell.

Read assembly and annotation: the informatic pipeline includes different steps, such as signal processing, base calling, alignment of reads to a reference genome and variant calling. The entire process is performed using appropriate analysis software for variant annotation. The performance of the sequencing run can be evaluated by analyzing different metrics, such as uniformity of base coverage, base coverage and on-target reads. 

**Figure 3:**
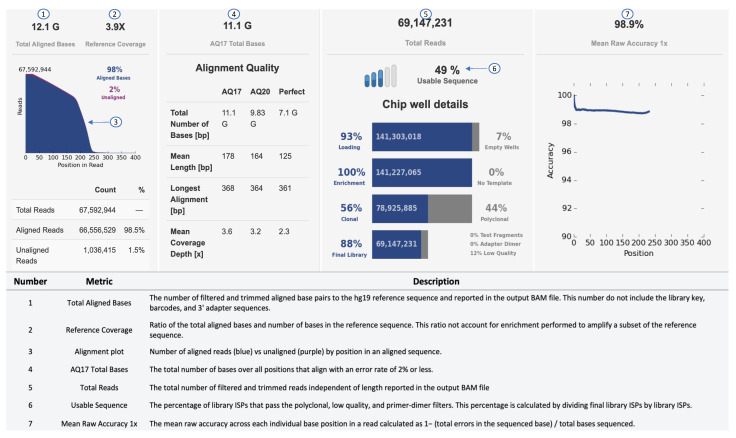
Sequencing performance of Ion Torrent. Metrics, such as Raw Accuracy, Aligned Bases, and Total Reads, can be used to determine the performance of a run.

An example of a sequencing run report of the Ion Torrent S5 sequencing output is shown in Figure 3. In the secondary analysis, variants can be filtered by different parameters, such as *p*-value (*p* < 0.001), phred quality score (p-read > 20), variant effect (missense, unknown, synonymous, InDels, SNVs), location (exon, intronic, splice-site, 5–3 UTR), Minor Allelic Frequency (MAF: 0.01–0.5) and allele frequency (40–60% for heterozygous; 90–100% for homozygous). To select disease-relevant pathogenic variants, effect prediction is performed using SIFT (Sorting Intolerant From Tolerant), PolyPhen (Polymorphism Phenotyping) or Fathmann score [100,101,102].

Considering the entire analytical NGS workflow (e.g., DNA isolation, library preparation and sequencing), the estimated cost for the analysis of HPA related genes is about EUR 120/sample (Table 3). This cost does not include equipment, labor or data analysis. The analysis of the HPA related genes using traditional methods would require higher costs and much longer times (Table 4).

## 7. Conclusions

HPA is the most commonly occurring amino acid metabolism genetic disorder characterized by serious clinical manifestations, including irreversible brain damage, intellectual deficiency and epilepsy. The precise and early diagnosis is remarkably successful in preventing these severe neurological features and ensuring healthy growth. Despite considerable progress having been made in the knowledge of this rare metabolic disorder, the diagnostic challenges are largely attributable to the marked clinical and genetic heterogeneity and the complexity of the Phe metabolic pathways involved, including additional unidentified key mediators with enzymatic, transporter, and regulatory functions. In this context, the advent of high-capacity and low-cost technologies and the use of *ad hoc* designed assays are producing a turning point for gene testing and clinical diagnosis of HPAs, improving our understanding of the basis of disease and the ability to better associate gene variants to specific phenotypes. The translation of fast, reliable and inexpensive genomic technologies into clinical practice will offer the opportunity for a better diagnosis of HPA in carrier patients, optimize clinical management, reduce the psychological burden and improve the development of early and effective therapeutic interventions.

## Figures and Tables

**Table 1:** Genetic causes of HPA.

Gene Symbol	Gene Name	Enzyme Function	Disease Name	OMIMnumber Disease	Mode of Inheritance	Variation Type	Variant Length	Molecular Consequence	Clinical Significance
*PAH*	Phenylalanine hydroxylase	Phe catabolism	Phenylketonuria, non-phenylketonuria, hyperphenylalaninemia	261600	AR	Deletion (131)	Less than 51 bp (1103)	Frameshift (97)	Conflicting interpretations (6)
						Duplication (32)	Between 51 and 1000 bp (8)	Missense (550)	Benign (65)
						Indel (6)	Between 1 and 50 kb (6)	Nonsense (64)	Likely benign (141)
						Insertion (37)	Between 50 and 500 kb (0)	Splice site (74)	Uncertain significance (220)
						Single Nucleotide (950)	Between 500 kb and 1 Mb (0)	ncRNA (0)	Likely pathogenic (265)
							Between 1 and 5 Mb (0)	Near gene (0)	Pathogenic (366)
							Greater than 5 Mb (3)	UTR (31)	
*DNAJC12*	DnaJ heat shock protein family (Hsp40) member C12	PAH folding and degradation	Hyperphenylalaninemia, mild, non-BH4-deficient	617384	AR	Deletion (8)	Less than 51 bp (45)	Frameshift (3)	Conflicting interpretations (0)
						Duplication (11)	Between 51 and 1000 bp (0)	Missense (11)	Benign (21)
						Indel (0)	Between 1 and 50 kb (1)	Nonsense (3)	Likely benign (4)
						Insertion (0)	Between 50 and 500 kb (0)	Splice site (6)	Uncertain significance (8)
						Single nucleotide (42)	Between 500 kb and 1 Mb (0)	ncRNA (0)	Likely pathogenic (4)
							Between 1 and 5 Mb (0)	Near gene (0)	Pathogenic (24)
							Greater than 5 Mb (6)	UTR (4)	
*GCH1*	GTP cyclohydrolase I	BH4 de novo biosynthesis	Hyperphenylalaninemia, BH4-deficient, B	233910	AR	Deletion (29)	Less than 51 bp (221)	Frameshift (12)	Conflicting interpretations (21)
						Duplication (25)	Between 51 and 1000 bp (3)	Missense (73)	Benign (36)
						Indel (2)	Between 1 and 50 kb (1)	Nonsense (8)	Likely benign (35)
						Insertion (16)	Between 50 and 500 kb (0)	Splice site (11)	Uncertain significance (89)
						Single nucleotide (197)	Between 500 kb and 1 Mb (0)	ncRNA (0)	Likely pathogenic (15)
							Between 1 and 5 Mb (1)	Near gene (0)	Pathogenic (70)
			Dystonia, DOPA-responsive, with or without hyperphenylalaninemia	128230	AD/AR		Greater than 5 Mb (4)	UTR (59)	
*PTS*	6-pyruvoyl-tetrahydropterin synthase	BH4 de novo biosynthesis	Hyperphenylalaninemia, BH4-deficient, A	261640	AR	Deletion (20)	Less than 51 bp (140)	Frameshift (7)	Conflicting interpretations (9)
						Duplication (10)	Between 51 and 1000 bp (1)	Missense (50)	Benign (14)
						Indel (0)	Between 1 and 50 kb (0)	Nonsense (5)	Likely benign (42)
						Insertion (3)	Between 50 and 500 kb (0)	Splice site (9)	Uncertain significance (36)
						Single nucleotide (127)	Between 500 kb and 1 Mb (1)	ncRNA (0)	Likely pathogenic (29)
							Between 1 and 5 Mb (0)	Near gene (0)	Pathogenic (41)
							Greater than 5 Mb (1)	UTR (5)	
*PCBD1*	Pterin-4-alpha-carbinolamine dehydratase	BH4 regeneration	Hyperphenylalaninemia, BH4-deficient, D	264070	AD/AR	Deletion (6)	Less than 51 bp (46)	Frameshift (2)	Conflicting interpretations (0)
						Duplication (11)	Between 51 and 1000 bp (0)	Missense (9)	Benign (13)
						Indel (0)	Between 1 and 50 kb (0)	Nonsense (4)	Likely benign (11)
						Insertion (1)	Between 50 and 500 kb (0)	Splice site (0)	Uncertain significance (14)
						Single nucleotide (41)	Between 500 kb and 1 Mb (1)	ncRNA (0)	Likely pathogenic (1)
							Between 1 and 5 Mb (0)	Near gene (0)	Pathogenic (18)
							Greater than 5 Mb (6)	UTR (14)	
*QDPR*	Quinoid dihydropteridine reductase	BH4 regeneration	Hyperphenylalaninemia, BH4-deficient, C	261630	AR	Deletion (21)	Less than 51 bp (123)	Frameshift (2)	Conflicting interpretations (2)
						Duplication (34)	Between 51 and 1000 bp (0)	Missense (27)	Benign (50)
						Indel (0)	Between 1 and 50 kb (0)	Nonsense (3)	Likely benign (17)
						Insertion (3)	Between 50 and 500 kb (0)	Splice site (4)	Uncertain significance (46)
						Single nucleotide (112)	Between 500 kb and 1 Mb (0)	ncRNA (65)	Likely pathogenic (9)
							Between 1 and 5 Mb (1)	Near gene (0)	Pathogenic (51)
							Greater than 5 Mb (13)	UTR (25)	

Abbreviations: AD, Autosomal dominant; AR, Autosomal recessive; OMIM, Online Mendelian Inheritance in Man; InDel, short insertions and deletions; UTR, Untranslated Regions.

**Table 2:** Advantages and challenges of various high-throughput methods.

	Targeted Gene Panel Sequencing	Whole-Genome/Exome Sequencing	CGH Microarray
Advantages	Higher coverage and sequencing depth	Massive parallel sequencing capability Identification of co-occurrence of mutations in different genes (all genes analyzed in parallel) Potential to identify new modifier genes/mutations Possibility to sequence non-coding variants and detect large insertion/deletion Quantitative and sensitive detection of genomic aberrations Constantly improving technology in gene capture and analysis	Identification of co-occurrence of CNVs in different genes Potential to identify new modifier genes/CNVs Quantitative and sensitive detection of genomic aberrations Relatively low costs Easy sample preparation Well-defined protocols and analysis pipeline Whole-genome analysis High resolution (up to 40 kb)
	Most suitable for clinical application		
	Higher number of samples in a single run		
	High degree of customizability		
	Reduced computational and storage resources		
	Low costs and turnaround time		
	Comprehensive sequencing of disease-associated regions (disease-specific scopus)		
	Single input of DNA/RNA		
	Identification of co-occurrence of mutations in different genes		
	Improved diagnostic rate (atypical phenotypes)		
	Decreased sequencing costs per gene		
	Constantly improving technology in gene capture and analysis		
Challenges	Selection of genes relevant for the disease Need for improved DNA variant database Genomic analysis restricted to selected regions (new genes cannot be identified) Requires thorough validation of assay performance as per guidelines Potential inclusion of non-validated genes in genetic testing (new variants of unknown significance in clinic) Selection of suitable target capture approach and sequencing platforms	Large amount of data Relatively complicated workflow and analysis Low number of samples Requires thorough validation of assay performance as per guidelines Computational costs and resources; consumable costs Informatic challenges for analysis and clinical reporting Long-term storage and retrieval of data Revalidation of upgrades (methodology rapidly changing) Coverage and data quality can vary across genes Limited applications in routine diagnostics	Low sensitivity and high background Annotations of probes CNVs of unknown significance in clinic Analysis of only pre-defined sequences Dynamic range limited by scanner Hybridization potentially non-specific

**Table 3:** NGS-targeted custom panels designed with AmpliSeqTM Designer Tool for Ion Torrent platform, and DesignStudio Sequencing Assay Designer for Illumina platform to analyze HPA related genes.

Gene Name	*QDPR*	*PTS*	*PCBD1*	*PAH*	*GCH1*	*DNAJC12*
Location	4p15.32	11q23.1	10q22.1	12q23.2	14q22.1–q22.2	10q21.3
Number of exons	7	6	6	13	7	6
Number of amplicons (Illumina)	10	8	5	17	11	8
Number of amplicons (Ion Torrent)	7	7	3	14	10	8
	Ion Torrent			Illumina		
Panel size	10.280 Kb			12.767 Kb		
Amplicon Range	101–234 bp			157–241 bp		
In silico coverage	100%					
Total Amplicons	49			59		
Run cost per sample (EUR )	119			110		

Instruments, GeneStudio-IonS5 (ThermoFisher Scientific, Waltham, MA, USA) and MiSeq System (Illumina Inc, San Diego, CA, USA); minimum coverage, 30×; sample source, germline.

**Table 4:** Comparison between next-generation sequencing and Sanger sequencing.

	Ion Torrent NGS-Based Method	Illumina NGS-Based Method	Sanger-Based Method
Data Generation	Millions of read/sample	Millions of read/sample	1 read/sample
Template preparation	Emulsion PCR	Cluster generation by bridge amplification	Chain termination/PCR
Accuracy	>99.0%	>99.0%	99.0%
Automation	High	High	Low
Nucleotide base per run	10–1.000 Mb	4 Mb–2 Gb	500 bp
	(based on the chip used)	(based on the chip/platform used)	
Read Length	Shorter	Shorter	Longer
	(<200 bases)	(<200 bases)	(300–800 bases)
Run costs	Cost-effective for a large number of targets		Cost-effective for a lower number of targets

## Data Availability

Not applicable.
